# The American Dental Association

**Published:** 1878-09

**Authors:** 


### THE AMERICAN DENTAL ASSOCIATION.

The annual meeting of this body was held at Niagara Falls on August 6th, and the three succeeding days.

The attendance, though not up to the usual number, was good, considering all things; there was about one hundred and ten present, and perhaps fifty dentists who were not members of the Association.

The time was very fully occupied; every standing committee, with, perhaps, one exception being prepared and reported, and upon some of the subjects there were three or four papers, nearly all of which were good. It was quite apparent that there had been during the year much studious investigation and thorough work in the domain of histology and physiology. The result of investigators in this direction must be pretty clearly stated and well established to stand under the criticism of *our* Dean, Atkinson and Black.

The work of all the standing committees was better done than ever before.

There was a large amount of routine and miscellaneous business transacted; much of this had reference to the proposed change by which sections were substituted for standing committees,

By this change it is presumed that the legitimate work of the body will be better done. The reports and papers will undergo supervision by the respective sections, and by that means additions, corrections and perhaps eliminations made which will render the reports more valuable.

In the following we present the action of the Association in view of the proposed change.

To carry out the object aimed at, the following changes and amendments were made in the Constition, viz:

ARTICLE III.

SEC. 3,—Insert the words "and membership of the sections" between the words "to office," at the end of the sixth line, and before the words "none being eligible," at the commencement of the seventh line.

ARTICLE V.

Sec. 6.—Strike out the sentence "they shall also report to the association upon any and all appliances which may be placed in their hands," near the bottom of the seventh page of the *printed* constitution.

ARTICLE V.

Sec. 6.—Page 8. Under head of "third division" strike out the words "names for Standing Committes for the ensuing year and" between the words "they shall report," and 'the names of places."

ARTICLE VI.

Strike out all five sections of Article vi, and insert a new Article as follows:

Sec. 1.—To prepare, arrange and expedite business, this Association shall be divided into six sections as follows:

1 —Artificial Dentistry, Metallurgy, and Chemistry.

2.—Dental Education.

3.—Dental Literature and Nomenclature.

4.—Operative Dentistry.

5.—Anatomy, Physiology, Histology, Microscopy, and Etiology.

6.—Pathology, Therapeutics, and Materia Medica.

Sec. 2.—It shall be the duty of each permanent member to inform the Recording Secretary, before the close of the morning session of the third day of the Annual Meeting, which of the six sections he elects to join for the ensuing year. He shall be free to follow his own preferences as to the particular channel in which he will labor, but can be a member of but one section during the same year.

Sec. 3.—It shall be the duty of the Recording Secretary immediately after the close of the above morning session of the third day, to make out lists of the names thus furnished of each section, as a basis for the election of officers of said sections.

Sec. 4.—The Association shall, by vote, assign some time after the regular election of officers of the Association, for the meeting of the several sections for organization.

Sec. 5.—When the time for organization of the sections, has arrived, it shall be the duty of the Recording Secretary to furnish some one member of each section with the list of members of his respective section, which member shall thereupon call the members to order, and read the list of names. The members shall then elect by ballot, a chairman and such other officers as they may see fit.

SEC. 6.—The chairman shall preside at all the meetings of his section. Shall exercise general supervision over the business, and shall see that the labors of his section are conducted in the manner calculated to secure the best results. The chairman, or such other member or members as the section shall select, shall prepare and read at the annual meeting of the Association next ensuing, a paper or papers on the advances and discoveries of the past year in the branches included in the section. The reading of such papers shall not together occupy more than forty minutes.

SEC. 7.—The several sections shall meet at ten a. m. on the morning of the first day of the annual meeting of the Association.

SEC. 8.—For the information of all members, a list of members of each section shall be published in the transactions of each year.

ARTICLE VIII.

Under "order of business" insert between Nos. one and two the words "two meetings of the sections at ten a. m. Change present No. two to three, and have it read "Organization of the Association at eleven a. m."

Change No. three to four.

Change No. four to five, and strike out the words "standing committes," and insert the word "sections."

Change Nos. six, seven, and eight to seven, eight, and nine.

Strike out all of No. nine.

After adoption of the above the various sections were formed, and each proceeded to organize for the work that devolved upon them.

The next number of the REGISTER will contain a synopsis of the proceedings of the meeting, in which the names of those constituting the sections will be given, and the organization of each.

